# Progenitor cell mobilisation and recruitment in pulmonary arteries in chronic obstructive pulmonary disease

**DOI:** 10.1186/s12931-019-1024-z

**Published:** 2019-04-16

**Authors:** Olga Tura-Ceide, Sandra Pizarro, Jéssica García-Lucio, Josep Ramírez, Laureano Molins, Isabel Blanco, Yolanda Torralba, Marta Sitges, Cristina Bonjoch, Victor I. Peinado, Joan Albert Barberà

**Affiliations:** 1 Department of Pulmonary Medicine, Hospital Clínic-Institut d’Investigacions Biomèdiques August Pi i Sunyer (IDIBAPS), University of Barcelona, Barcelona, Spain; 2 Department of Thoracic Surgery, Hospital Clínic-Institut d’Investigacions Biomèdiques August Pi i Sunyer (IDIBAPS), University of Barcelona, Barcelona, Spain; 3 Department of Cardiology, Hospital Clínic-Institut d’Investigacions Biomèdiques August Pi i Sunyer (IDIBAPS), University of Barcelona, Barcelona, Spain; 4 Department of Pathology, Hospital Clínic-Institut d’Investigacions Biomèdiques August Pi i Sunyer (IDIBAPS), University of Barcelona, Barcelona, Spain; 5 Biomedical Research Networking Center on Respiratory Diseases (CIBERES), Madrid, Spain; 6 Biomedical Research Networking Center on Cardiovascular Diseases (CIBERCV), Madrid, Spain; 7 Servei de Pneumologia, Hospital Clínic, Villarroel, 170, 08036 Barcelona, Spain

**Keywords:** COPD, Endothelial dysfunction, Progenitor cells, Vascular remodeling, DLco

## Abstract

**Background:**

Pulmonary vascular abnormalities are a characteristic feature of chronic obstructive pulmonary disease (COPD). Cigarette smoking is the most important risk factor for COPD. It is believed that its constant exposure triggers endothelial cell damage and vascular remodelling. Under pathological conditions, progenitor cells (PCs) are mobilized from the bone marrow and recruited to sites of vascular injury. The aim of the study was to investigate whether in COPD the number of circulating PCs is related to the presence of bone marrow-derived cells in pulmonary arteries and the association of these phenomena to both systemic and pulmonary endothelial dysfunction.

**Methods:**

Thirty-nine subjects, 25 with COPD, undergoing pulmonary resection because of a localized carcinoma, were included. The number of circulating PCs was assessed by flow cytometry using a triple combination of antibodies against CD45, CD133 and CD34. Infiltrating CD45^+^ cells were identified by immunohistochemistry in pulmonary arteries. Endothelial function in systemic and pulmonary arteries was measured by flow-mediated dilation and adenosine diphosphate-induced vasodilation, respectively.

**Results:**

COPD patients had reduced numbers of circulating PCs (*p* < 0.05) and increased numbers of CD45^+^ cells (< 0.05) in the pulmonary arterial wall than non-COPD subjects, being both findings inversely correlated (r = − 0.35, *p* < 0.05). In pulmonary arteries, the number of CD45^+^ cells correlated with the severity of vascular remodelling (r = 0.4, *p* = 0.01) and the endothelium-dependent vasodilation (r = − 0.3, *p* = 0.05). Systemic endothelial function was unrelated to the number of circulating PCs and changes in pulmonary vessels.

**Conclusion:**

In COPD, the decrease of circulating PCs is associated with their recruitment in pulmonary arteries, which in turn is associated with endothelial dysfunction and vessel remodelling, suggesting a mechanistic link between these phenomena. Our findings are consistent with the notion of an imbalance between endothelial damage and repair capacity in the pathogenesis of pulmonary vascular abnormalities in COPD.

**Electronic supplementary material:**

The online version of this article (10.1186/s12931-019-1024-z) contains supplementary material, which is available to authorized users.

## Background

Chronic obstructive pulmonary disease (COPD) is a highly prevalent, non-curable, life-threatening disease, characterised by irreversible changes in lung structures [1–3]. Pulmonary vascular abnormalities, as shown by alterations in vessel structure, abnormal cell growth, endothelial dysfunction and resistance to apoptosis, are characteristic features in COPD [4, 5]. An altered pulmonary vascular function in COPD patients may predispose to pulmonary hypertension, which is associated with adverse outcomes [6]. Growing evidence suggests that endothelial cell damage found in pulmonary vessels of COPD patients is an initial and important triggering factor that promotes pulmonary vascular remodelling [5]. It has been shown that COPD patients present endothelial dysfunction in both pulmonary and systemic arteries at early disease stages [7–9]. This endothelial damage may result from an imbalance between vascular injury and the body’s repair capacity [10].

Bone marrow-derived progenitor cells (PCs) are a population of rare, pre-differentiated adult stem cells that circulate in the blood with the ability to proliferate and differentiate into mature endothelial cells [11]. Although, the contribution of PCs in vascular remodelling and repair is unclear, it is believed that under pathological conditions, PCs are mobilized from the bone marrow and recruited to sites of vascular injury to maintain vascular homeostasis [7]. PCs are believed to be essential in maintenance of the endothelium’s integrity and restoration of normal function, replacing terminally differentiated cells lost as a consequence of physiological cell turnover or tissue damage [12]. Conversely, it has also been suggested that in COPD, PCs mobilization and recruitment may contribute to COPD pathogenesis. PCs intrinsic dysfunctional activity, mainly due to cigarette smoke (CS) exposure, promotes inflammation, pulmonary vessel remodelling and pulmonary hypertension [13].

Reduced number and function of circulating PCs has been established as an independent prognostic factor associated with endothelial dysfunction, high cardiovascular risk and increased mortality [14–16]. In COPD, few studies to date have investigated the number of circulating PCs [10, 12, 17]. We and others have previously shown reduced levels of circulating PCs in COPD patients, as compared to controls [10, 12, 17]. To date, two different mechanisms have been suggested to explain the reduced number of circulating PCs seen in COPD, an impairment of the bone marrow to generate enough circulating PC numbers and/or an increased PC recruitment in pulmonary vessels in response to tissue injury. Accordingly, in this study we aimed to quantify in the same individuals both circulating PCs and bone marrow-derived cells localized in pulmonary arteries, as well as their association to pulmonary artery remodelling, and systemic and pulmonary artery functionality.

## Methods

### Subjects

Thirty-nine subjects, aged between 48 and 70 years, undergoing pulmonary resection because of a localized lung carcinoma were enrolled in the study. Patients were divided into two groups: (a) a non-COPD group (*n* = 14) with normal pulmonary function, and (b) a COPD patient group (*n* = 25), defined by a forced expiratory volume in the first second (FEV_1_)-to-forced vital capacity (FVC) ratio lower than 0.7. Patients with COPD were on regular bronchodilator treatment and some of them received inhaled corticosteroids. Before surgery, all patients underwent standard evaluation by means of medical history, clinical examination and pulmonary function tests (forced spirometry, lung volumes, carbon monoxide diffusing capacity (DLco), and arterial blood gas analysis), as previously described [18]. Plasma levels of endothelin-1, nitrites/nitrates, angiopoietin-2 and brain natriuretic peptide (BNP) were also measured. The study was conducted in accordance with the Declaration of Helsinki, approved by the institutional Committee on Human Research and all subjects gave written informed consent.

### Circulating CD45^+^CD34^+^CD133^+^progenitor cells

The number of circulating progenitor cells was evaluated by flow cytometry using antibodies against CD45 (pan-leukocyte marker), CD133 (sub-population of haematopoietic stem cells) and CD34 (mature and progenitor endothelial cells) as previously described [10, 17, 19]. In brief, mononuclear cells (MNCs) were isolated by Ficoll density gradient separation, washed once with phosphate buffered saline (PBS) supplemented with 2% foetal calf serum (FCS) and resuspended at 2 × 10^6^ cells (control tube) and at 4 × 10^6^ cells (sample tube). Peripheral blood mononuclear cells were stained and analysed for phenotypic expression of surface markers using pre-conjugated anti-human monoclonal antibodies: anti-CD45-FITC, anti-CD34-PECy7 and anti-CD133-PE. The fluorescence minus one technique [10, 17, 20] was employed to provide negative controls. After 45 min of incubation, cells were washed, resuspended into 500ul of PBS + 2%FCS and proceeded to flow-cytometric analysis. Eighty thousand events were gated in the lymphocyte region as preciously described [10, 17].

### Endothelial function in systemic and pulmonary arteries

#### Systemic endothelial function assessment

Endothelial function was assessed by high resolution ultrasound as the change in brachial artery diameter in response to reactive hyperaemia (flow-mediated dilation), as previously described [21]. Endothelium-independent, nitroglycerine-mediated dilation was also measured.

#### Endothelial pulmonary artery function assessment

Endothelial function of pulmonary artery segments was evaluated in vitro as previously described [4, 22]. Arterial segments with an external diameter of approximately 2 mm were carefully dissected free of visible fat and connective tissue and cut into 3 mm long rings. All rings were submaximally pre-contracted with norepinephrine (NE; 10^− 7^ to 10^− 6^ M) to obtain a stable plateau of tension. Rings were tested using cumulative concentrations of adenosine diphosphate (ADP) (10^− 10^ to 10^− 4^ M), an endothelium-dependent vasodilator. Relaxation of each pulmonary artery ring was determined by measuring the reduction in tone in response to cumulative doses of the vasodilating agent and expressed as the percent reduction from the value recorded after pre-contraction with NE. Maximal relaxation was the greatest reduction in tone in response to the vasodilator.

### Number of CD45^+^ cells in pulmonary arteries

Cryostat sections of PBS-4% paraformaldehyde-fixed artery rings were immunostained using the avidin-biotin complex/horseradish peroxidase method (Vector Laboratories). We used a mouse monoclonal antibody against the pan-leukocyte marker CD45 (diluted 1/750) (Novocastra Laboratories) and a progenitor cell marker CD133 (diluted 1/5) (Miltenyi Biotec) for immunolocalization of bone marrow-derived cells. Negative control experiments were conducted omitting the primary antibody. The number of positive cells infiltrating the arterial wall were counted and expressed as cell number per square millimetre of intimal surface as previously described [22].

### Morphometric studies

Pulmonary muscular arteries were analysed in formalin-fixed paraffin-embedded lung tissue sections processed with elastic orcein stain. All arteries with an external diameter < 1 mm and with complete elastic laminas were evaluated using a specific software package (Leica-Qwin) as previously described [23]. Briefly, the external diameter was measured as the widest distance between external elastic laminas, perpendicular to the greatest longitudinal axis of each artery. Arterial wall thickness of each pulmonary artery was calculated as the area of the arterial wall divided by the internal perimeter and expressed as percentage of radius. All arteries with an index of narrowing (smaller diameter divided by larger diameter) lower than 1/3 were rejected.

### Data analysis

Data are expressed as mean ± SD. Comparisons between groups were performed using Mann-Whitney U-test. Spearman rank correlation was used to assess correlations between variables. A *p* value < 0.05 was considered statistically significant.

## Results

### General patient’s characteristics

The COPD and non-COPD groups were well matched with respect to age and body mass index. Both groups showed a high percentage of male subjects. Five patients in the non-COPD group had never smoked. Approximately, half of the patients in each group were current smokers. All COPD patients were current or ex-smokers (Table 1). COPD patients had moderate-to-severe airflow limitation, moderately reduced DLco and mild hypoxemia. There were no differences in the Framingham risk score between groups. The number of leukocytes, monocytes, lymphocytes and neutrophils were similar in both groups. COPD patients presented lower platelet counts than non-COPD subjects (Table 1). Levels of endothelin-1, angiopoietin-2, C-reactive protein, fibrinogen, nitrites/nitrates, VEGF, IL-6, BNP and VEGR2 did not differ between groups (Table 1).

**Table 1:** Clinical characteristics, lung function and laboratory measurements

	Non-COPD	COPD
Age, years	57.3 ± 7.2	58.5 ± 5.8
Male sex, n (%)	9 (64.2%)	24 (96%)
Body mass index (Kg/m^2^)	24.6 ± 1.7	25.4 ± 2.8
Never smoked, %	5 (35.7%)	0 (0%)
Current smokers, %	6 (43%)	17 (68%)
Ex-smokers, %	3 (21%)	8 (32%)
Smoking history, pack-years	38.6 ± 22.4	60.1 ± 33.9
Expiratory carbon monoxide, ppm	1.5 ± 1.5	2.4 ± 2.2
FEV_1,_ % predicted	91.7 ± 10.5	56.7 ± 21.5***
FEV_1_/FVC, %	76.2 ± 4.5	51.0 ± 14.7***
TLC, % predicted	90.9 ± 14.5	106.6 ± 17.3*
RV, % predicted	108.8 ± 20.8	164.4 ± 69.2**
DL_CO,_ % predicted	82.5 ± 11.9	63.4 ± 20.1**
PaO_2,_ mmHg	85.0 ± 12.8	75.3 ± 10.5 *
PaCO_2,_ mmHg	37.3 ± 2.8	37.3 ± 3.8
Total cholesterol, mg/dL	196.0 ± 31.4	203.2 ± 39.5
Triglycerides, mg/dL	92.4 ± 25.0	111.2 ± 46.7
HDL, mg/dL	47.0 ± 12.3	48.6 ± 12.1
LDL mg/dL	129.4 ± 28.0	132.4 ± 34.0
Framingham risk score^$^	8.4 ± 4.8	10.9 ± 5.7
Glucose, mg/dL	97.1 ± 10.3	100.7 ± 20.5
Leukocyte count, × 10^9^/L	7.7 ± 1.7	8.4 ± 1.5
Lymphocyte count, × 10^9^/L	1.6 ± 0.5	1.8 ± 0.4
Monocyte count, × 10^9^/L	0.4 ± 0.1	0.4 ± 0.1
Neutrophils, ×10^9^/L	5.2 ± 1.5	5.7 ± 1.4
Red blood cells, × 10^12^/L	4.6 ± 0.4	4.6 ± 0.3
Haemoglobin, g/L	137.0 ± 13.2	146.5 ± 9.3*
Platelet count, × 10^9^/L	309.5 ± 88.0	252.2 ± 61.0*
C-reactive protein, mg/L	1.1 ± 1.5	1.0 ± 2.1
Fibrinogen, mg/L	5.4 ± 1.9	4.0 ± 1.2
Nitrites/nitrates, nMol/mL	20.4 ± 7.0	22.4 ± 10.6
Endothelin-1, pmol/L	6.5 ± 4.3	5.8 ± 2.3
Angiopoietin-2, pg/mL	527.0 ± 99.6	433.3 ± 142.0
VEGF, pg/mL	49.8 ± 41.0	57.8 ± 78.7
VEGFR2, pg/mL	34.4 ± 10.4	53.7 ± 7.8
IL-6, pg/mL	5 ± 7.2	7.7 ± 16.0
BNP. pg/mL	15.9 ± 6.2	21.8 ± 18.2

Definition of abbreviations: *COPD* chronic obstructive pulmonary disease, *DLco* lung diffusing capacity for carbon monoxide, *RV* residual volume, *FEV*_*1*_ forced expiratory volume in 1 s, *FVC* forced vital capacity, *TLC* total lung capacity, *PaCO*_*2*_ partial pressure or arterial carbon dioxide, *PaO*_*2*_ partial pressure of arterial oxygen. Framingham risk score^$^ can range from −6 to 19, with higher scores indicating greater cardiovascular risk. Non-COPD (*n* = 14), COPD (*n* = 25), * *p* < 0.05, ** *p* < 0.01, *** *p* < 0.001, compared with non-COPD, Mann Whitney test. Values expressed as mean ± SD

### COPD patients showed lower number of circulating CD45^+^CD34^+^CD133^+^cells and greater number of CD45^+^ cells in pulmonary arteries than non-COPD subjects

**Fig. 1:**
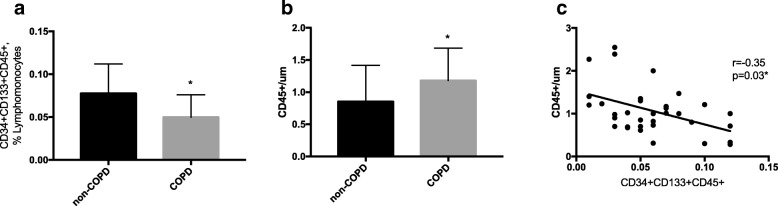
Number of CD45^+^CD34^+^CD133^+^ and CD45^+^ cells in COPD. **a** Number of circulating CD34^+^CD133^+^CD45^+^ progenitor cells in non-COPD and COPD patients expressed as percent of lymphomonocytes; (**b**) Quantification of CD45^+^ infiltrates in the intima of the pulmonary arteries expressed as a number per mm of endothelium in non-COPD and COPD subjects, * *P* < 0.05, compared with non-COPD subjects, Mann Whitney test. Values expressed as mean ± SD; (**c**) Relationship between the number of CD45^+^cells and the number of circulating CD45^+^CD34^+^CD133^+^ cells; Spearman rank correlation test, Non-COPD (*n* = 13), COPD (*n* = 24), * *P* < 0.05

Circulating progenitor cells, defined as CD45^+^CD34^+^CD133^+^cells were significantly reduced in COPD patients compared with the non-COPD group (Fig. 1a). Conversely, COPD patients had a higher number of CD45^+^ cells in pulmonary arteries than non-COPD subjects (Fig. 1b). Representative histological pictures showed CD45^+^and CD133^+^ cells localized within the intima of pulmonary arteries (Additional file 1: Figure S1a-b). There was a significant inverse correlation between the number of circulating CD45^+^CD34^+^CD133^+^ cells and the number of CD45^+^ infiltrating the intima of the pulmonary arteries (r = − 0.35, *p* = 0.03) (Fig. 1c).

### Endothelial function in systemic and pulmonary arteries

#### Systemic endothelial function assessment

COPD patients and non-COPD subjects showed similar values for flow mediated dilation (FMD) (1.8 ± 1.0 vs 1.6 ± 1.7). FMD was unrelated to the number of circulating CD45^+^CD34^+^CD133^+^ cells (r = − 0.2, *p* = 0.3) or to the presence of CD45^+^cells in the intima of pulmonary arteries (r = − 0.1, *p* = 0.4).

#### Endothelial pulmonary artery function assessment

Endothelium-dependent dilation of isolated pulmonary arteries induced by ADP and distensibility were reduced in COPD patients compared to non-COPD subjects (Table 2). There was no significant correlation between systemic FMD and pulmonary endothelial function or between systemic FMD and distensibility (Table 2). The number of CD45^+^ infiltrating the artery wall of pulmonary arteries was inversely correlated with endothelial function (r = − 0.3, *p* = 0.05) and distensibility (r = − 0.4, *p* = 0.04), whereas the number of circulating CD45^+^CD34^+^CD133^+^ cells did not.

**Table 2:** Endothelial function and distensibility of pulmonary arteries

	Non-COPD	COPD
ADP-induced vasodilation, % change from maximal contraction	−88.2 ± 26.0	−83.5 ± 22.6
Distensibility, vol/mmHg	2.0 ± 0.8	1.4 ± 0.5*

Definition of abbreviations: *ADP* adenosine diphosphate. Non-COPD (*n* = 11), COPD (*n* = 23), Mann Whitney test. Values expressed as mean ± SD. * *p* < 0.05

### Morphological evaluation

**Fig. 2:**
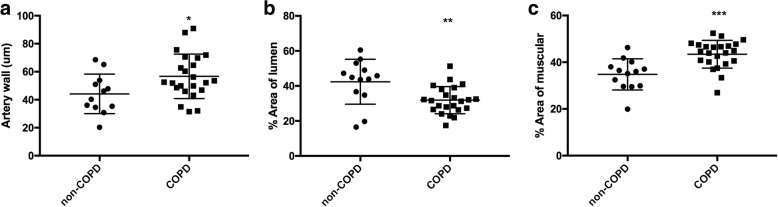
Morphometric measurements in non-COPD and COPD subjects. **a** Percentage of artery wall thickness, % measured radius; (**b**) Percentage of lumen area, % total area; (**c**) Percentage of muscular area, % total area in non-COPD and COPD subjects. Non-COPD (*n* = 13), COPD (*n* = 23), * *P* < 0.05, ** *P* < 0.01compared with non-COPD subjects, Mann Whitney test. Values expressed as mean ± SD

Morphometric measurements of pulmonary arteries are shown in Table 3. COPD patients had thicker artery walls (Fig. 2a), reduced lumen area (Fig. 2b), thicker muscular layer and a trend to thicker intima than non-COPD subjects (Fig. 2c).

**Table 3:** Morphometric measurements on pulmonary arteries

Variables	Non-COPD	COPD
Mean wall thickness (μm)	44.1 ± 14.1	56.6 ± 15.8*
Measured external diameter (μm)	210.7 ± 61.0	252.3 ± 120.5
Calculated external diameter, um	538.4 ± 147.9	531.1 ± 117.2
Index of narrowing, %	73.5 ± 3.9	72.8 ± 8.1
Wall thickness, % measured diameter	34.0 ± 9.6	41.0 ± 16.5
Intimal area, % total area	22.8 ± 8.9	24.7 ± 7.2
Muscular area, % total area	32.8 ± 8.9	43.4 ± 5.9***
Lumen area, % total area	42.3 ± 12.8	31.8 ± 7.7**

Definition of abbreviations: *COPD* chronic obstructive pulmonary disease; Non-COPD (*n* = 9), COPD (*n* = 23), * *p* < 0.05, ** *p* < 0.01, *** *p* < 0.001 compared with non-COPD, Mann Whitney test. Values are expressed as mean ± SD

**Fig. 3:**
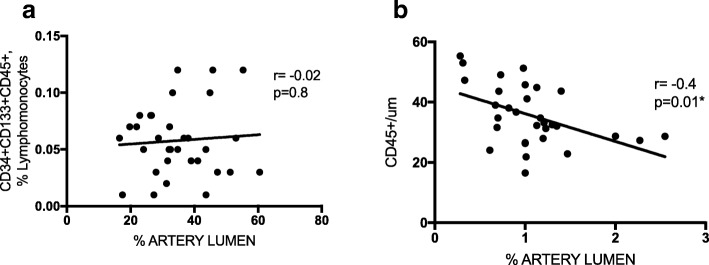
CD45^+^CD34^+^CD133^+^, CD45^+^ cells and the percentage of artery lumen. **a** Relationship between the number of CD45^+^CD34^+^CD133^+^ cells and the percentage of lumen area, % total area artery lumen of pulmonary arteries; (**b**) Relationship between the number of CD45^+^ cells and the percentage of lumen area, % total area artery lumen of pulmonary arteries. Non-COPD (*n* = 11), COPD (*n* = 21), Spearman rank correlation test, * *P* < 0.05

Whereas the number of CD45^+^ localized in the intima of the pulmonary arteries inversely correlated with artery lumen area, the number of circulating CD45^+^CD34^+^CD133^+^ cells did not (Fig. 3a, b).

### Relationships among progenitor cells, pulmonary vascular remodelling, pulmonary function and CS exposure

**Fig. 4:**
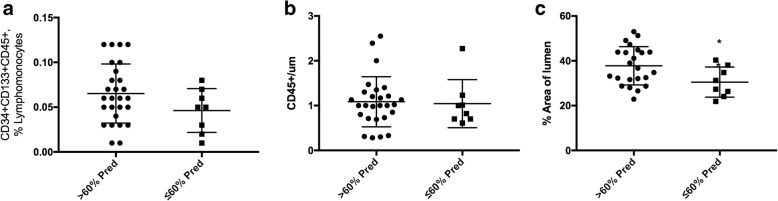
CD34^+^CD133^+^CD45^+^, CD45^+^ cells and % of artery lumen and DLco levels. **a** Number of circulating CD34^+^CD133^+^CD45^+^progenitor cells in non-COPD and COPD patients grouped according to DLco above or below the median value (60% predicted); (**b**) Number of CD45^+^cells in non-COPD and COPD subjects grouped according to DLco above or below the median value (60% predicted); (**c**) % of artery lumen in non-COPD and COPD subjects grouped according to DLco above or below the median value (60% predicted). Non-COPD (*n* = 11), COPD (*n* = 21), * *P* < 0.05, Mann Whitney test. Values expressed as mean ± SD

In COPD patients, the number of circulating PCs or bone marrow-derived cells localized in pulmonary arteries was unrelated to the severity of airflow obstruction or DLco values (Fig. 4a, b). Yet, COPD patients with DLco values below the median value had greater pulmonary vascular remodelling, as shown by lower lumen area, than those with DLco values above the median value (Fig. 4c). Non-COPD controls were separated in non-smokers and smokers and compared to COPD patients (Additional file 2: Table S1). Smokers and COPD subjects showed a higher percentage of male subjects than non-smoker controls. In most parameters analyzed, non-COPD smokers presented an intermediate phenotype between non-smoker controls and COPD subjects (FEV_1,_ % predicted; FEV_1_/FVC, %; DL_CO,_ % predicted; PaO_2,_ mmHg; CD45^+^CD34^+^CD133^+^ cells; mean wall thickness (um); lumen area, % total area and muscular area, % total area) (Additional file 2: Table S1).

## Discussion

The present study shows that in COPD the decrease of circulating PCs is associated with the presence of bone marrow-derived cells in pulmonary arteries and the number of CD45^+^ cells infiltrating the intima of pulmonary arteries directly correlated with pulmonary vascular remodelling.

Circulating CD45^+^CD34^+^CD133^+^ PCs were significantly reduced in COPD patients compared with the non-COPD group, confirming recent findings published by our group and others [10, 12, 17]. Reduced number of circulating PCs has been established as a prognostic risk factor associated with endothelial dysfunction and increased cardiovascular risk [14]. PCs residing in the bone marrow under homeostatic conditions can be mobilized into the circulation in response to tissue damage and are believed to be essential in tissue regeneration replacing terminally differentiated cells lost because of physiological cell turnover or tissue damage [10].

The role of PCs in COPD and tissue regeneration is not fully understood, and different mechanisms have been suggested to explain the reduced number of circulating PCs seen in COPD patients. One potential explanation is an increased recruitment of these cells to sites of tissue injury, namely in the pulmonary vasculature. Tissue repair and regeneration following injury is thought to involve resident cell proliferation as well as a selective recruitment of circulating progenitor cell populations through complex signalling cascades [24]. Our group has previously shown an increased number of CD133^+^ progenitor cells localized in the wall of pulmonary arteries of COPD patients [22]. Similarly, in this study a significant number of CD133^+^ progenitor cells localized within the pulmonary artery of COPD patients suggesting their involvement in pulmonary vessel repair mechanisms.

In the present study, we extend these previous findings and show that COPD patients present a greater number of CD45^+^ cells in pulmonary arteries than non-COPD subjects. Interestingly, the number of CD45^+^ cells within the pulmonary artery wall was inversely correlated with the number of circulating CD45^+^CD34^+^CD133^+^ cells, patients with lower number of circulating PCs had a higher number of CD45^+^ cells in the vessel wall. Although this correlation does not establish a cause and effect relationship, it is plausible that in COPD, progenitor cell mobilization and homing in pulmonary arteries may occur in response to vascular damage, causing the depletion of the pool of circulating PCs.

Exposure to CS is the primary cause of COPD and plays a key role in PC dysfunction [25]. Dysfunctional PCs show excessive apoptosis and are unable to respond to external injuries to mediate proper lung tissue healing [26]. Previous results from our group showed in an experimental animal model, that short-term exposure to CS induced PCs dysfunction, affecting pulmonary homing and proliferation [27]. In vitro, CS altered the rate of proliferation, senescence, differentiation and migration capacity of PCs [27]. In COPD patients, dysfunctional PCs may be unable to support the normal repair of the pulmonary vasculature promoting neointima formation, vessel remodelling and disease progression. In line with these results, our group has previously shown that bone marrow derived CD133^+^ cells had the capacity to migrate from the vessel lumen into the intima and differentiate into smooth muscle cells, exerting remodelling effects on injured vessels [28].

In the present study, COPD patients displayed thicker pulmonary artery walls and reduced lumens than non-COPD subjects. Interestingly, the increased number of CD45^+^ cells localized in the pulmonary artery wall was associated with a reduction in the arterial lumen. This is in agreement with previous observations from our group showing that structural alterations in pulmonary arteries occur at early stages in COPD and that the number of CD133^+^ cells attached to the endothelium was greater in COPD patients than in control subjects [23]. Overall, the current and previous findings suggest a potential causative role of progenitor cell recruitment in the pathogenesis of pulmonary vascular remodelling in COPD.

COPD patients and non-COPD subjects showed similar values of systemic endothelial function, though both groups had lower values than those previously observed in healthy non-smokers [17]. This indicates that both COPD patients and non-COPD subjects in this study presented systemic endothelial dysfunction. We have previously reported significant differences in FMD values between control non-smokers and both COPD patients and smokers without COPD [17]. Accordingly, the reduced value of FMD shown herein in the non-COPD group is likely due to the fact that most of the non-COPD subjects were heavy smokers. When the non-COPD group was divided in non-smokers and smokers a marked increase of FMD was seen in the non-smoker group compared to the smoker control group. We also observed that FMD values were unrelated to the number of circulating PCs or CD45^+^ cells localized in the pulmonary artery wall. This agrees with previous findings where no significant correlation was found between the number of circulating PCs and FMD values between COPD and non-COPD subjects [17].

Endothelium-dependent dilation and distensibility values measured in isolated pulmonary arteries were reduced in COPD patients compared to non-COPD subjects and correlated with the number of infiltrating CD45^+^ cells in pulmonary arteries. This is consistent with the notion that impairment of the endothelial function in pulmonary arteries may promote the mobilization and homing of bone marrow-derived PCs. Systemic endothelial dysfunction did not correlate with pulmonary endothelial dysfunction. We consider that endothelial dysfunction in systemic arteries is more likely due to chronic CS exposure rather than a systemic effect of COPD. On the other hand, pulmonary endothelial dysfunction appears to be more directly related to progenitor cell homing in response to injury and vessel remodelling. Overall, these findings suggest that CS might exert direct effects on endothelial function in both systemic and pulmonary arteries, irrespective of the number of recruited PCs in remodelled pulmonary arteries.

DLco values were not associated with variations in the number of circulating PCs or bone marrow-derived cells recruited in pulmonary vessels. Yet, lower DLco values were associated with greater remodelling of pulmonary arteries. Neither the severity of airflow obstruction nor the values of PaO_2,_ were related to changes in PCs or pulmonary vascular remodelling. Overall, our findings are in line with the notion that DLco is a marker of pulmonary vascular integrity. Finally, non-COPD controls were separated in non-smokers and smokers and compared to COPD patients. In most parameters analyzed, non-COPD smokers presented an intermediate phenotype between non-smoker controls and COPD subjects indicating the key role of CS exposure in lung and endothelial dysfunction.

The main strength of this study was the simultaneous measurement of markers of vascular integrity and function in both systemic and pulmonary arteries, using lung tissue samples, in the same individual. Nevertheless, our study has some limitations. First, the reduced number of subjects, which is inherent to the need to evaluate patients undergoing lung resection in whom the lung neoplasm was localized and did not produce changes in lung parenchyma or respiratory function. Second, the CD45 maker used in tissue assessment is not specific to progenitor cells and could also indicate recruitment of other cell populations. Further studies using double staining for both CD133 and CD45 immunofluorescence are required to prove that these migrated cells in the intima of the pulmonary arteries are derived from circulating PCs. Third, in this study we could not be certain that the number of CD45^+^ cells present in the pulmonary walls and the reduced artery lumen area, were directly associated to the reduced number of circulating CD45^+^CD34^+^CD133^+^ cells in these patients. However, the direct correlation between the number of circulating CD45^+^CD34^+^CD133^+^ cells and the number of CD45^+^ infiltrates in pulmonary vessels and the presence of CD133^+^ cells, an intrinsic marker of progenitor cells, observed in the vessel wall might infer that in COPD patients, circulating CD45^+^CD34^+^CD133^+^ cells are recruited in response to injury to the pulmonary vessel wall, causing a reduction in circulating PCs numbers.

Interestingly, recent data suggested the existence of a “vasculogenic zone” in the wall of human blood vessels, which might serve as a reservoir for PCs capable to differentiate into mature endothelial cells [29]. Accordingly, the presence of PCs recruited as a result of an injury response could not solely be explained by the homing of bone-marrow derived circulating PCs as tissue resident PCs could also play an important role. Finally, some of the results are represented as correlations and therefore only describe an association, they do not prove a cause and effect relationship.

## Conclusion

This integrative study shows a reduction of circulating PCs in COPD, which is associated with the recruitment of bone marrow-derived cells into the patient’s pulmonary artery wall. This, in turn, is associated with endothelial dysfunction and vessel remodelling of pulmonary arteries. Overall, our results suggest the contribution of bone marrow-derived PCs in pulmonary vascular remodelling and are consistent with the notion of an imbalance between endothelial damage and repair capacity in the pathogenesis of pulmonary vascular abnormalities in COPD.

## Additional files

Additional file 1: **Figure S1.** Immunolocalization of CD45^+^ and CD133^+^cells in pulmonary arteries. (a) Representative micrograph of a transversal section of a pulmonary artery (around 2 mm diameter) stained with a monoclonal antibody against CD45 (arrows are showing positive cells in the intima layer). (b) Representative micrograph of a transversal section of a pulmonary artery stained with a monoclonal antibody against CD133 (arrows are showing positive cells in the intima and sometimes in the media layer). (PDF 706 kb)

Additional file 2: **Table S1.** Clinical characteristics, lung function, endothelial function, PCs numbers and morphometric measurements. (DOCX 16 kb)

## Abbreviations

ADP: Adenosine diphosphate
BNP: Brain natriuretic peptide
COPD: Chronic obstructive pulmonary disease
CS: Cigarette smoke
DLco: Lung diffusing capacity for carbon monoxide
FEV_1_: Forced expiratory volume in 1 s
FMD: Flow mediated dilation
FVC: Forced vital capacity
PaCO_2_: Partial pressure or arterial carbon dioxide
PaO_2_: Partial pressure of arterial oxygen
PC: Progenitor cells
RV: Residual volume
TLC: Total lung capacity
