# Supplementary figures and images for: Progenitor cell mobilisation and recruitment in pulmonary arteries in chronic obstructive pulmonary disease
Source: Respir Res. 2019 Apr 16;20:74. doi: 10.1186/s12931-019-1024-z (PMC6469212; doi:10.1186/s12931-019-1024-z)

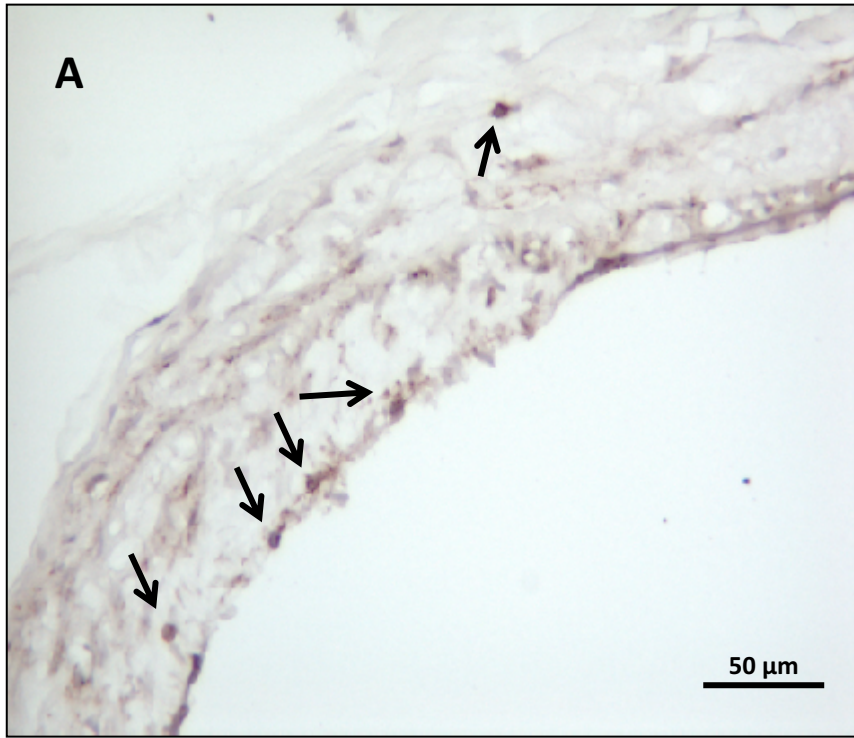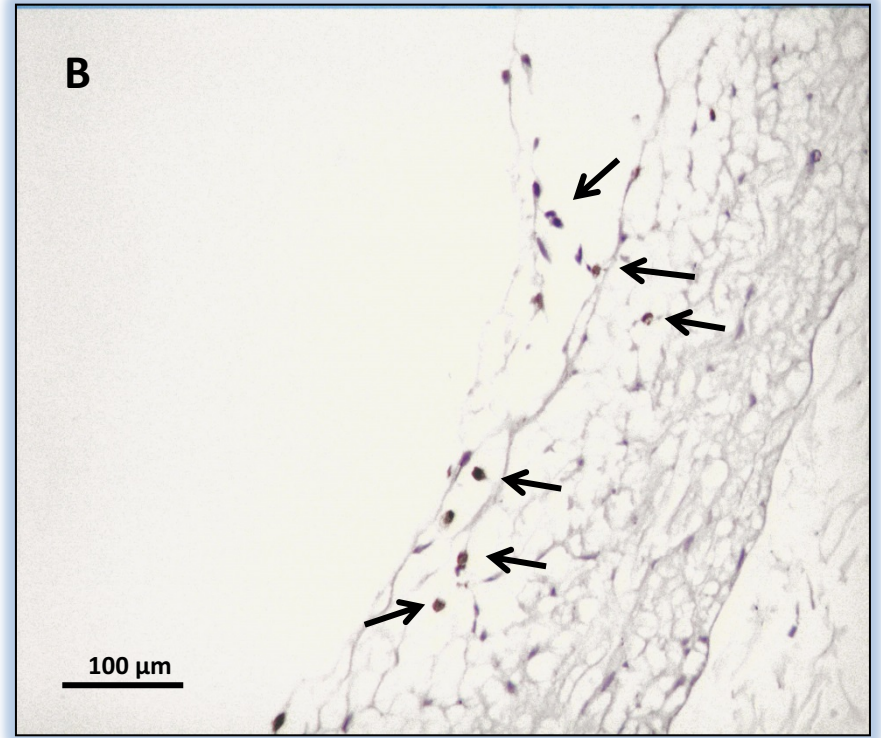

Supplement: Supplementary file 1 — Figure S1. Immunolocalization of CD45+ and CD133+cells in pulmonary arteries. (a) Representative micrograph of a transversal section of a pulmonary artery (around 2 mm diameter) stained with a monoclonal antibody against CD45 (arrows are showing positive cells in the intima layer). (b) Representative micrograph of a transversal section of a pulmonary artery stained with a monoclonal antibody against CD133 (arrows are showing positive cells in the intima and sometimes in the media layer). (PDF 706 kb) [file 12931_2019_1024_MOESM1_ESM.pdf]
