# Supplementary material for: Progenitor cell mobilisation and recruitment in pulmonary arteries in chronic obstructive pulmonary disease
Source: Respir Res. 2019 Apr 16;20:74. doi: 10.1186/s12931-019-1024-z (PMC6469212; doi:10.1186/s12931-019-1024-z)
Supplement: Supplementary file 2 — Table S1. Clinical characteristics, lung function, endothelial function, PCs numbers and morphometric measurements. (DOCX 16 kb) [file 12931_2019_1024_MOESM2_ESM.docx]

**Supplementary Materials:**

|  | **Non-Smokers**  **N=5** | **Smokers**  **N=9** | **COPD**  **N=25** |
| --- | --- | --- | --- |
| Age, years | 58.7 ± 7.5 | 56.3 ± 7.6 | 58.5 ± 5.8 |
| Male sex, n (%) | 2 (40%) | 7 (77.7%) | 24 (96%)* |
| Body mass index (Kg/m^2^) | 24.7 ± 2.3 | 24.6 ± 1.4 | 25.4 ± 2.8 |
| Smoking history, pack-years | 0 ± 0 | 37.3± 21.4**^$^** | 60.1 ± 33.9* |
| Expiratory carbon monoxide, ppm | 0.8 ± 0.8 | 2.2 ± 2.2 | 2.4 ± 2.2 |
| FEV_1,_ % predicted | 105.4 ± 8.9 | 85.6 ± 3.8**^$^** | 56.7 ± 21.5*† |
| FEV_1_/FVC, % | 78.8 ± 5.2 | 73.8 ± 6.9 | 51.0 ± 14.7*† |
| TLC, % predicted | 98.2 ± 6.0 | 92.3 ± 11.4 | 106.6 ± 17.3† |
| DL_CO,_ % predicted | 88.0 ± 13.9 | 80.8 ± 11.5 | 63.4 ± 20.1*† |
| PaO_2,_ mmHg | 94.8 ± 16.7 | 77.5± 17.0 | 75.3 ± 10.5* |
| PaCO_2,_ mmHg | 38.0 ± 4.3 | 36.4 ± 2.7 | 37.3 ± 3.8 |
| CD45^+^CD34^+^CD133^+^ cells | 0.09 ± 0.0 | 0.07±0.0 | 0.05 ± 0.02* |
| CD45^+^ cells | 1.16 ± 0.8 | 0.7± 0.3 | 1.2 ± 0.5† |
| Flow-mediated dilation (FMD) | 3.2 ± 2.2 | 0.9± 1.2 | 1.8± 1.0 |
| Distensibility, vol/mmHg | 2.0 ± 0.4 | 2.0 ± 0.9 | 1.4 ± 0.5† |
| ADP-induced vasodilation,  % change from max. contraction | -79.2 ± 30.5 | -87.9 ± 27.3 | -83.5 ± 22.6 |
| Mean wall thickness (um) | 39.6 ± 20.4 | 43.2 ± 13.8 | 56.6 ± 15.8† |
| Lumen area, % total area | 44.5 ± 19.6 | 41.4 ± 9.9 | 31.8 ± 7.7† |
| Muscular area, % total area | 29.9 ± 8.2 | 34.1 ± 9.3 | 43.4 ± 5.9*† |

**Supplementary table 1. Clinical characteristics, lung function, endothelial function, PCs numbers and morphometric measurements.**

Definition of abbreviations: COPD, chronic obstructive pulmonary disease; DLco, lung diffusing capacity for carbon monoxide; FEV_1_, forced expiratory volume in 1s; FVC, forced vital capacity; TLC, total lung capacity; PaCO_2,_ partial pressure or arterial carbon dioxide; PaO_2_, partial pressure of arterial oxygen; ADP, adenosine diphosphate. Non-COPD non-smoker (n=5), non-COPD smoker (n=9) and COPD (n=25), ^$^ p<0.05, between smokers and non-smoker controls; *p<0.05, between non-smokers vs COPD and †p<0.05 between smokers’ vs COPD. Mann Whitney test. Values expressed as mean±SD.
